# The role of host autophagy machinery in controlling *Toxoplasma* infection

**DOI:** 10.1080/21505594.2018.1518102

**Published:** 2018-09-29

**Authors:** Sébastien Besteiro

**Affiliations:** DIMNP, UMR5235 CNRS, Université de Montpellier, Montpellier, France

**Keywords:** *Toxoplasma gondii*, toxoplasmosis, autophagy, non-canonical autophagy, LC3-associated phagocytosis (LAP)

## Abstract

*Toxoplasma gondii* is an obligate intracellular parasitic protist that infects a wide range of warm-blooded vertebrates. Although this parasite can cause serious complications, infections are often asymptomatic, allowing *T. gondii* to persist in its host and possibly enhancing the chances of its transmission. *T. gondii* has thus evolved multiple mechanisms of host manipulation to establish chronic infection. This persistence involves a balance between host immunity and parasite evasion of this immune response. This review highlights recent investigations that have demonstrated the important role played by the autophagy machinery in this balance, both in parasite control by the host, and in host exploitation by the parasite.

## Introduction

Approximately one-third of the world’s population is infected by *Toxoplasma gondii*, a parasitic protist belonging to the phylum Apicomplexa. Several factors make this parasite so successful. First, this intracellular pathogen has a wide host range and is able to invade any warm-blooded animal. Second, the infection is usually largely asymptomatic and the parasite is able to survive in its host throughout its whole life, providing more opportunities for transmission. Felids such as domestic cats are the only known definitive hosts in which the parasite can undergo sexual replication. All other non-feline warm-blooded vertebrates can act as intermediate hosts, where *T. gondii* replicates asexually. In intermediate hosts (including humans), *T. gondii* is present as actively dividing tachyzoites responsible for the symptoms of a disease called toxoplasmosis[1], or more quiescent bradyzoites which are found in tissue cysts[2].

Upon primary infection of an immunocompetent host, an acute phase of the disease is initiated by the actively-replicating tachyzoites. Yet, as it is usually controlled by the immune system of the host, it often remains asymptomatic and will be followed by a chronic phase whereby the parasites will differentiate into encysted bradyzoites[3]. In the case of immune suppression (i.e. for HIV/AIDS patients, cancer patients with chemotherapy and transplant recipients), uncontrolled bradyzoites can reactivate into tachyzoites whose fast replication will lead to devastating tissue destruction and potentially serious illnesses (i.e. encephalitis, pneumonitis, chorioretinitis or myocarditis). Another severe form of the disease is during pregnancy, through a transplacental transmission of the parasite from a primary infected mother to a developing foetus. As with many pathogens, the severity of the infection is variable and depends on several factors influencing the interplay between host and parasite, such as the type of host, its immune status, but also the type of parasite strain. For instance, severe ocular disease or even death, are possible in immunocompetent individuals infected with highly virulent strains of *T. gondii*. [4]

Although *T. gondii* is the only species described in the *Toxoplasma* genus, there are different strains with some degree of genetic variation. The majority of strains isolated in Europe and North America belong to one of three clonal lineages (referred to as types I, II and III)[5], however there is a much greater diversity in other parts of the world, especially South America[6]. Several of these strains differ markedly in virulence in the mouse model: type I is considered the most virulent and lethal, while types II and III are considered avirulent and are associated with chronic infection[7].

## Immune response to *T. gondii* in mice and in humans

**Figure 1.**
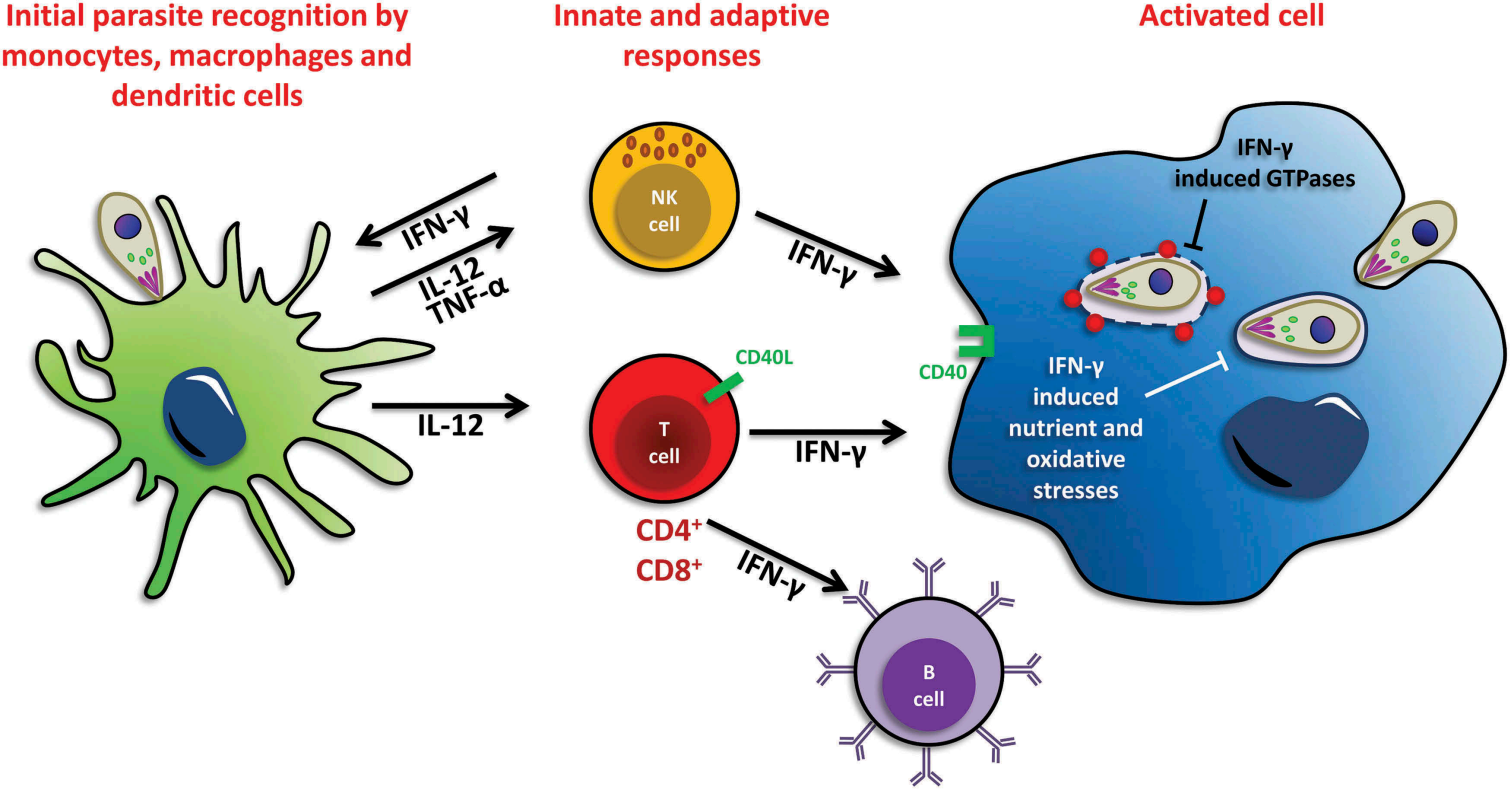
**Schematic view of immune responses to *T. gondii* upon initial infection**. Cells involved in the innate and adaptive immune response to *T. gondii* infection are shown. Initial host control of parasite infection induces the production of the pro-inflammatory cytokine interleukin 12 (IL-12) by macrophages and dendritic cells. IL-12 will in turn activate natural killer (NK) and T cells to secrete interferon γ (IFN-γ). Neutrophils and T cells also produce IFN-γ in response to infection. IFN-γ then activates several host defense mechanisms for intracellular elimination of *T. gondii*, including the activation of interferon-induced GTPases, and the induction of nutrient and oxidative stresses. Activated B cells can also help limiting the spread of the parasites to some extent.

A large number of studies on *T. gondii* infection have been performed in mice, which are natural hosts of the parasite and excellent models for studying innate and adaptive immune responses[8]. Although there are differences, the immune response in human hosts follows a similar general pattern. Early in infection, tachyzoites can induce a strong innate immune response (Figure 1). The general paradigm is that sentinel cells of the mononuclear phagocyte system (monocytes, macrophages, dendritic cells) are activated by the parasites. In turn they will activate an adaptive immune response mediated by B and T lymphocytes [9] through the action of pro-inflammatory cytokines such as IL-12[10]. Innate lymphoid cells such as Natural Killer cells are also critical to license antigen-presenting cells and produce soluble factors such as interferon gamma (IFN-γ) that contribute to parasite control[11]. Ultimately, various mechanisms of cell-autonomous immunity triggered by CD8 T cell activity and Th1-polarized CD4 T cell responses are important for parasite control[12], as well as to some extent the humoral response through B cells[13]. This essentially constrains tachyzoites and enhances their differentiation to bradyzoites confined to tissue cysts, which will remain largely hidden from the immune system. It should be noted that there are, however, some differences between humans and mice, for example in the mechanisms triggering the innate cytokine response[14], or in some effectors of the cell-autonomous immunity[15].

Tachyzoites infect their host cells by an active invasion process [16] leading to the formation of a specialized membrane-bound compartment called the parasitophorous vacuole (PV) that is not destroyed by fusion with the host endo-lysosomal system[17]. Control of acute infection by this parasite stage depends largely on IFN-γ and associated pathways[18], in both mice [19,20] and humans [21,22]. It should be noted, however, that although it is commonly admitted that IFN-γ-dependent pathways are pivotal in the control of toxoplasmosis, other molecular signals such as the cytokine tumor necrosis factor alpha (TNF-α) [23] and the CD40 (a member of the tumor necrosis factor receptor superfamily)/CD40 ligand interaction [24–26] also play a significant role, although this may be more the case in humans than mice. IFN-γ mediates host protection via multiple mechanisms, including the induction of antimicrobial molecules (ie nitric oxide and reactive oxygen species) and is also responsible for changes in host metabolism that limit parasite replication (Figure 1)[27]. Moreover, in mice and humans it induces immunity-related GTPases (IRGs) and guanylate-binding proteins (GBPs) that can lead to the destruction of parasite-containing PVs ^28^,(Figure 1). However, the efficiency of this depends largely on parasite strains: types II and III parasites are for example susceptible to the IRGs, while highly virulent type I parasites can secrete kinase-like proteins that inactivate these GTPases.^29,30^ Overall, this shows that *T. gondii* has successfully developed strategies to modulate host immune effectors to ensure a delicate balance between parasitism and the host’s immune response. As parasites ultimately depend on their host for survival and transmission, they both have to preserve it and also remain hidden from the immune system to avoid destruction. For instance, some parasite effectors promote a pro-inflammatory microenvironment to protect the host from excessive parasite growth, while others are protecting *T. gondii* from the mounting immune response [31,32].

## The host autophagy machinery participates in the control of intracellular pathogens

Autophagy is a self-digestive lysosomal degradation pathway found in most eukaryotic cells. The most widespread autophagic process is macroautophagy, which is generally simply referred to as “autophagy” (I shall do likewise in this review). It relies on the formation of a double membrane structure called the autophagosome that will engulf intracellular material and subsequently fuse with lysosomes for degradation and recycling of its content[33]. Autophagy is particularly important for providing sources of energy in response to nutrient stresses, but it also plays a housekeeping role in removing misfolded or aggregated proteins and clearing damaged organelles[34]. Finally, autophagy can be involved in the elimination of intracellular pathogens by a process called “xenophagy” (“xenos” meaning “stranger” in Greek, as in that case it leads to the disposal of material of foreign origin) [35,36].

**Figure 2.**
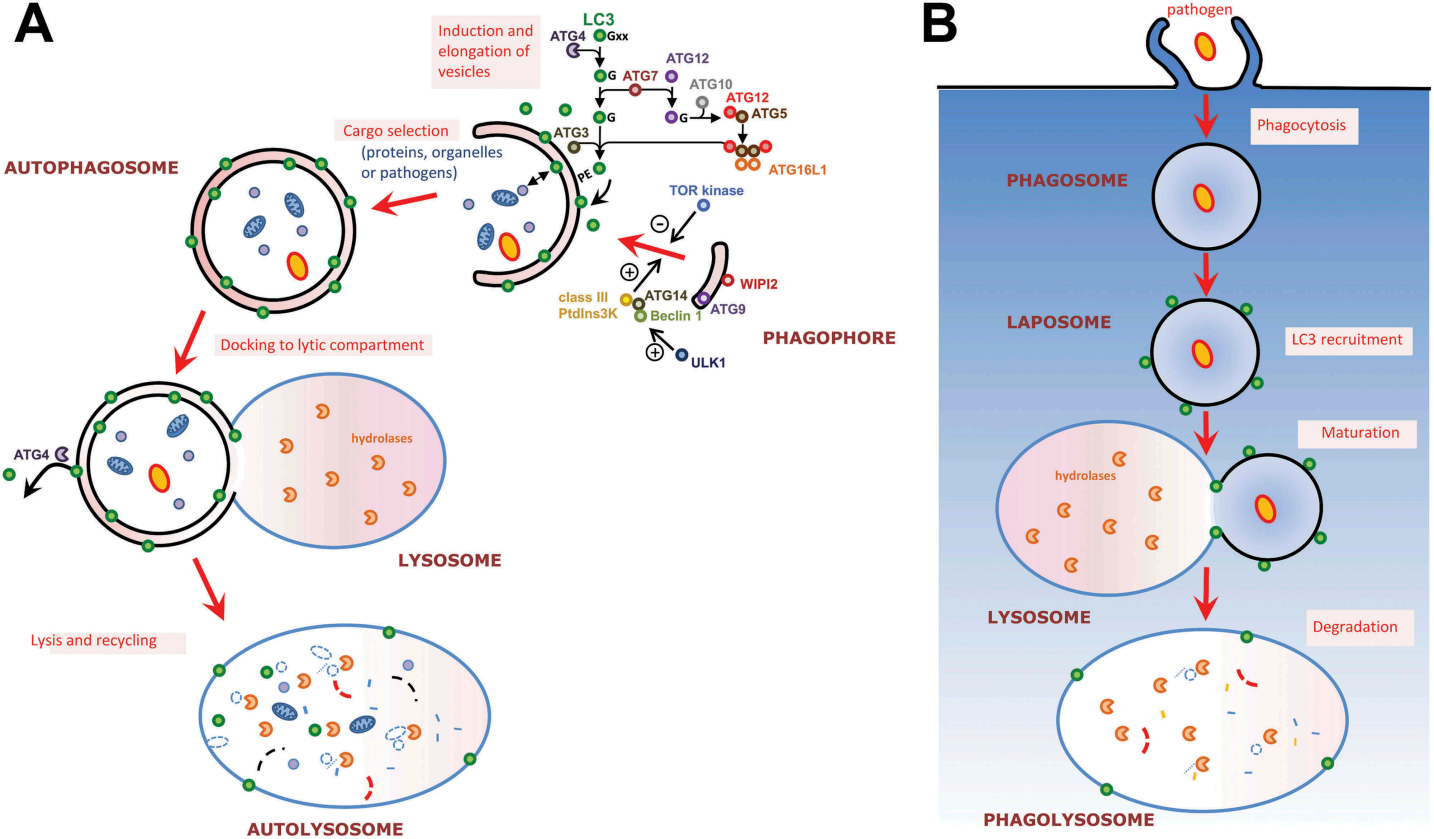
**Autophagy-related protein LC3 associates with different types of membranes. (a)** Schematic representation of the mammalian autophagic process and its regulatory machinery. The process is initiated by the formation of a structure called the phagophore, which engulfs cytoplasmic cargo and, once complete, will form a double membrane compartment called the autophagosome. The autophagosome will then fuse with lysosomes to form an autolysosome where the autophagic cargo will be degraded for subsequent recycling. The whole process is regulated by upstream kinases such as Target of Rapamycin (TOR) and class III phosphatidylinositol 3-kinase (PtdIns3K). Microtubule-associated protein 1 Light Chain 3 (LC3), an important player for autophagosome formation, is conjugated to phosphatidyl ethanolamine (PE) on the membrane of elongating phagophores thanks to ubiquitin-like conjugation systems. **(b)** The process of LC3-associated phagocytosis (LAP) is involved in the degradation of extracellular pathogens. LC3 associates with phagosomal membranes thanks to the same ubiquitin-like conjugation machinery required for LC3 membrane conjugation to the phagophore during canonical autophagy. The emerging phagosome, now referred to as LAPosome, will mature by fusion with lysosomes and the cargo will be degraded.

The formation of the autophagosome is a highly regulated process depending on a molecular machinery[33], of which a large part has been identified thanks to mutant screening assays in the yeast *Saccharomyces cerevisiae*[37]. AuTophaGy-related (ATG) proteins coordinate this process, which is initiated by the formation of a double-membrane cytosol-sequestering vesicle, termed the phagophore that can engulf either random portions of the cytosol or specific proteins and organelles (Figure 2(a)). This is regulated by upstream kinases such as the Target Of Rapamycin (TOR) complex, acting as a repressor[38], and the class III phosphatidylinositol 3-kinase (PtdIns3K) complex, which is a positive regulator[39]. The phagophore then matures into a fully closed autophagosome that will subsequently fuse with lysosomes to give a hybrid organelle called autolysosome, in which digestion and recycling of the sequestered material will take place (Figure 2(a)).

One central protein in the process is a ubiquitin-like regulator of autophagosome biogenesis called microtubule-associated protein 1 Light Chain 3 (LC3)[40]. LC3 association to the autophagosomal membrane is tightly regulated by a core ubiquitin-like conjugation machinery that consists of proteins ATG3, 4, 5, 7, 10, 12 and 16L1 (Figure 2(a)). Briefly, the C-terminus of LC3-I (the cytosolic form of the protein) is cleaved by the ATG4 protease to expose a glycine residue. This residue is then covalently linked to the membrane-embedded lipid phosphatidylethanolamine, though the action of a ubiquitin-like conjugation cascade including ATG7, ATG3, and ATG12/ATG5-ATG16L1 acting as E1, E2, and E3 enzymes, respectively. The resulting membrane-bound form is called LC3-II. It should be noted that in mammalian cells, there are at least six homologues, classified into the LC3/GABARAP subfamilies, that can potentially be conjugated to the autophagosomal membrane in a similar way[41], but might play different roles in the autophagic process[42].

Interestingly, a non-canonical recruitment of LC3 to single-membrane phagosomes surrounding intracellular pathogens has been described recently and termed LC3-Associated Phagocytosis (LAP, Figure 2(b))[43]. Recruitment of LC3 to phagosomes occurs rapidly (≤ 15 min) after internalization and is transient[44]. The formation of these LC3-decorated phagosomes (also called LAPosomes) involves several components of the canonical autophagy machinery. A PtdIns3K complex (although different from the one involved in regulating canonical autophagy) is for instance important for LAP initiation[45], by generating PtdIns3P that will act as a signaling platform at the phagosome membrane. The two ubiquitin-like conjugation systems required for LC3 membrane conjugation during canonical autophagy (ATG7, ATG3, and ATG12/ATG5-ATG16L1), are also required for the conjugation of LC3 to the LAPosome[45]. As for autophagosomes, phagosomes (and LAPosomes) have to fuse with lysosomes to mature as lytic compartments and subsequent degrade their cargo. LC3 function on LAPosomes is not fully elucidated, but it seems it could accelerate their maturation by fusion with lysosomes [45,46]. Rab7 and its interacting partners on endosomes and phagosomes are however also able to promote fusion with lysosomes[47], but how LC3 coordinates with other known regulators of phagosome maturation is currently unknown.

## IFNγ-dependent parasite clearance is mediated by the autophagy machinery independently of autophagosome formation

**Figure 3.**
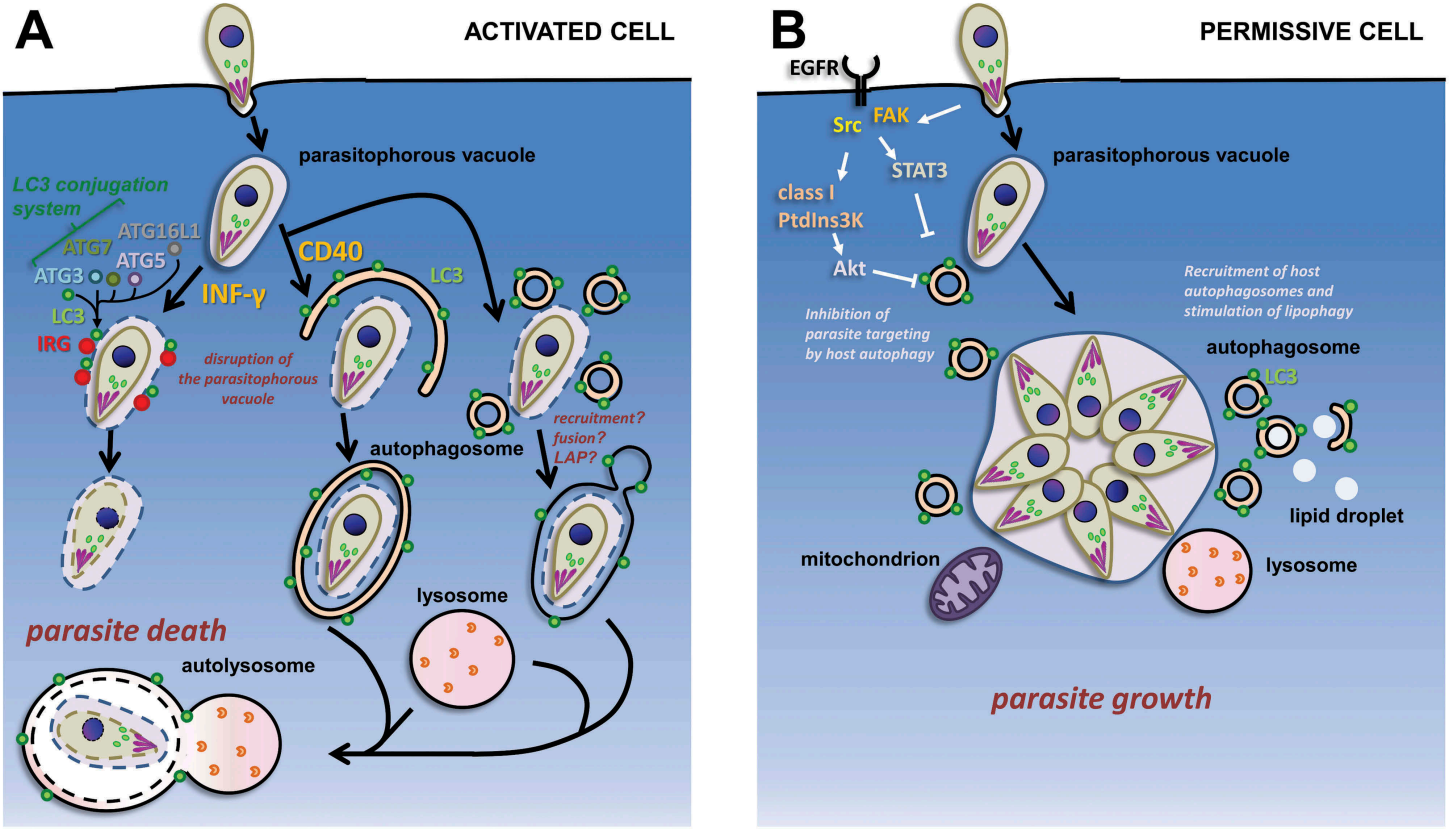
**Schematic representation of the interplay between host autophagy-related machinery and intracellular tachyzoites. (a)** Possible autophagy proteins-dependent pathways for the degradation of tachyzoites in immunologically-activated mouse host cells. The LC3-centered autophagy machinery has an IFN-γ-stimulated non-canonical role in the recruitment of immunity-related GTPases (IRGs) to the parasitophorous vacuole; on the other hand, CD40 stimulation leads to parasite elimination by canonical autophagy, LAP, or a related lysosome-dependent mechanism. (**b)** In permissive host cells, invading tachyzoites can potentially block the autophagy machinery of the host by interfering with epidermal growth factor receptor (EGFR)-dependent signaling pathways. Developing parasites can also recruit host organelles, including autophagic vesicles, to access nutrients sources.

In the mouse model (Figure 3(a)), killing of *T. gondii* inIFN-γ-activated host cells is associated with blebbing, vesiculation and ultimately stripping of the PV membrane early after cell entry. The recruitment of IRGs to the PV membrane leads to its destruction, exposure of the parasite to the host cytoplasm and its subsequent elimination, although the precise mechanism by which the IRG proteins achieve this is not known[48]. Initial observations in activated macrophages described autophagosome-like double membrane vacuoles surrounding entire parasites, suggesting a role for autophagy in the elimination of the tachyzoites[49]. Others have also described an accumulation of LC3-positive vesicles near the disrupted PV in activated host cells before their elimination, however the authors did not see structures resembling autophagic vacuoles engulfing whole parasites by cryo-electron microscopy[50]. Moreover, during canonical autophagy the double-membrane-bound autophagosome normally goes on to fuse with LAMP1-positive lysosomes, but degraded *T. gondii*-containing vacuoles are not uniformly LAMP1-positive [50,51]. Finally, proteins that are important for the initiation of autophagosome formation, like ATG9, or upstream regulators ATG14 and Beclin 1 (Figure 2(a)), are dispensable for parasite destruction [52–54]. It thus appears IFN-γ-treated cells can kill parasites through a mechanism that is independent of the formation of canonical autophagosomes.

However, intriguingly, not only LC3 is recruited to the vicinity of PVs, but functional studies have shown parasite elimination depends on autophagy proteins which are important for LC3 lipidation. More precisely, in mice the recruitment of IRG proteins to the PV membrane and subsequent destruction of the parasite is promoted by ATG5 [51,55], a key protein involved in LC3 conjugation to membranes (Figure 2(a)). Interestingly, knockout of ATG5 in macrophages and neutrophils not only increases susceptibility to infection by *T. gondii*, but also by the bacterium *Listeria monocytogenes*[51]. Besides, IFN-inducible GTPases have been implicated in cellular immunity against bacteria via the autophagy machinery, thus suggesting it is a general defense mechanism against intracellular pathogens [28,56]. Although ATG5 is clearly involved in the control of the parasites, autophagosomal membranes were not observed around intracellular tachyzoites[51], again suggesting that canonical autophagy is not at play here. In dendritic cells, ATG5 may even modulate the CD4 + T cell cytokine response through mechanisms different from canonical autophagy, but also distinct from LC3-associated phagocytosis or the IRG-dependent response[57].

Importantly, not only ATG5, but the entire LC3 conjugation system, including ATG7, ATG3, and the ATG5-ATG12-ATG16L1 complex, are involved in IFN-γ-dependent anti-*T. gondii* IRG recruitment (Figures 2(a) and 3(a)) [52,53,58]. This suggests the presence of PV-located LC3 is the main factor that recruits the IFN-inducible GTPases specifically to the target membrane. Interestingly, not only LC3, but also its homologs of the GABARAP family localize to the membrane of the parasite-containing PV in a conjugation-dependent manner and are able to recruit IFN-γ-induced GTPases[59]. It should be noted that there does not seem to be a complete functional redundancy between these different members of the LC3 family, as GABARAPs might be more important for parasite control[54]. Moreover, how LC3 and related proteins recruit the GTPases to the PV is currently unclear[60].

The aforementioned studies have been essentially performed in mice and although human cells express a wide repertoire of GBPs, they do not necessarily express the IRGs found in mice[61]. Besides, human GBPs are thought to be either dispensable for IFN-γ–induced inhibition of *T. gondii* proliferation, [52] or to be acting without being targeted to the PV[62]. However, it is interesting to note that proteins of the LC3-conjugating machinery such as ATG16L1 (Figure 2(a)) are also important for the recruitment of human GBPs to parasite-containing vacuoles[52].

## Cd40-dependent parasite clearance mediated by the autophagy machinery

The CD40-dependent parasite elimination may represent an important complementary mechanism to the primary IFN-γ-mediated pathways[63]. Interestingly, there is an IFN-γ-independent/CD40-dependent autophagy machinery-related killing of intracellular tachyzoites[64]. In contrast with IFN-γ-dependent killing of *T. gondii*, the CD40-stimulated pathway promotes killing of the parasites by the lysosomal system (Figure 3(a))[64]. Moreover, canonical autophagy seems involved, as CD40 signaling appears to act on autophagy. More precisely, it acts on LC3 recruitment around the parasites [65] through the stimulation of upstream regulators such as Beclin 1[63], a regulatory subunit of autophagy-promoting PtdIns3K (Figure 2(a)), and ULK1[66], a kinase that regulates this complex (Figure 2(a))[67]. In non-hematopoietic cells, the down-regulation of autophagy proteins (Beclin 1 or ATG7), or the use of the PtdIns3K-inhibitor 3-methyladenine to block autophagy, can also prevent the CD40-dependent fusion between LC3-decorated *T. gondii*-containing vacuoles and the lysosomes, and inhibit subsequent killing of the parasites[65]. Altogether, this shows that the CD40-induced anti-*T. gondii* activity depends on proteins of the autophagy machinery, but in contrast to IFN-γ-mediated killing, it seems to involve the sequestration and degradation of intracellular tachyzoites through classical degradative autophagy.

However, although a double membrane structure was noted around the *T. gondii*-containing PV in autophagy-inducing conditions[68], it is unclear how autophagosomes could be built to accommodate such large objects (autophagosomes of mammalian cells are usually < 1.5 µm in diameter)[69]. Instead of a single autophagosome that would surround whole parasite-containing PVs, the LC3-positive structures could represent local recruitment and fusion of multiple autophagosomes (Figure 3(a)). It could also be a non-canonical recruitment of LC3 directly to the PV membrane, similar to the LAP process (Figure 3(a)). However, LAP is known to be dependent on Beclin 1 and the PtdIns3K complex[45], but independent of ULK 1 activity[70].

## *T. gondii* can block and also exploit host autophagy

In parallel to the implication of host autophagy in the control of intracellular microbes, there is also a growing list of pathogens that seem to be able to antagonize the host autophagy machinery, or even exploit it to enhance their replication[36]. Similarly, there is some evidence *T. gondii* is activating host cell signaling that counter-regulates the autophagy machinery, in order to avoid degradation. [68,71] Establishment of the parasites in a PV leads to a molecular cascade preventing the targeting of *T. gondii* by the autophagy machinery through activation of epidermal growth factor receptor (EGFR)-dependent pathways (Figure 3(b)). Parasite-dependent activation of EGFR and a downstream effector PtdIns3K-regulated Akt pathway, prevents parasite targeting for autophagic/lysosomal degradation in endothelial, epithelial and glial cells[68]. Akt is a regulator of the TOR kinase, a critical modulator of autophagy induction[34]. Parasite-activated EGFR-dependent signaling is also acting on the functions of signal transducer and activator of transcription 3 (STAT3) that can regulate autophagy via the transcriptional regulation of several genes, or can inhibit autophagy by sequestering the eukaryotic Initiation Factor 2 alpha (eIF2α)[72].

To support its growth, *T. gondii* is also known to be very efficient at intercepting host organelles shortly after invasion and its establishment in the PV. These are probably used as a source of nutrients, in particular lipids[73]. They include the endoplasmic reticulum and mitochondria[74], as well as vesicles of the endolysosomal pathway [75,76], several Rab-associated vesicles[77], and lipid droplets (Figure 3(b)) [78,79].

Interestingly, a study performed in HeLa cells and primary fibroblasts presents evidence that *T. gondii* is able to induce a significant recruitment of the autophagy machinery in these cells, as highlighted by LC3-positive vesicles and Beclin 1 localization around the PV[80]. This recruitment of autophagosomes seems to be independent of TOR, but parasite growth was reduced in ATG5-deficient cells, indicating a beneficial role for host cell autophagy in the development of the parasites. The recruitment of autophagosomes was observed several hours after invasion and is similar to the recruitment of other host cell organelles. More recently, it was shown that infection by *T. gondii* triggers lipophagy (the autophagy of host lipid droplets) to provide a source of fatty acids for parasite development[81]. Altogether this suggests that in non- IFN-γ-activated cells, host cell autophagy is exploited by the parasite for acquiring nutrients to sustain its growth (Figure 3(b)).

## Conclusion and outstanding questions

Overall, the current knowledge indicates a significant, but still not completely elucidated, role of members of the autophagy machinery in eliminating *T. gondii* in IFN-γ-primed host cells. Upon stimulation by IFN-γ, the autophagy machinery regulating the membrane association of proteins of the LC3 family with parasite-containing PVs, allows the recruitment of GTPases for the disruption of the PV membrane and elimination of the parasites. This process is clearly different from canonical autophagy/xenophagy as it seems largely independent from classical upstream regulators of the autophagic pathway, and does not seem to directly involve lysosomal degradation of the parasites. A number of questions remain regarding this original role for the autophagy-related machinery at parasite-containing PVs. First, mammalian cells express a number of LC3 homologues, and as currently described in the literature their respective contributions to this particular process are not completely clear [54,59]. Also, at the PV membrane it is not known if these proteins of the LC3 family can directly act as a scaffold for the GTPases, or as a signal in conjunction with IFN-γ activation for further recruitment of currently unknown protein partners. Ubiquitin and p62 (a selective autophagy adaptor with a ubiquitin-binding domain) are also recruited to the PV in an IFN-γ-dependent way and are likely to also play an important role for the recruitment of GTPases [82–85].

As mentioned above, a significant part of the studies on parasite control by the autophagy machinery of the host has been performed in the mouse model, and mostly in cells of the mononuclear phagocyte system. They demonstrated a prominent role played for IRGs in the IFN-γ-driven response, but different mechanisms are also potentially playing a significant role, noticeably in other hosts or other cell types. For instance, an IFN-γ-independent/CD40-dependent upregulation of the autophagy machinery and recruitment of LC3 to *T. gondii*-containing vacuoles for its elimination has also been characterized in both mice and human cells[64]. In contrast with the IFN-γ GTPase-mediated pathway, this depends on well-characterized upstream regulators of the autophagic process and intersects with the lysosomal pathway, which is suggestive of a canonical degradative autophagy. However, the nature of the LC3-decorated structures surrounding the parasites is incompletely characterized. The recent discovery of LAP suggests that the observation of LC3 around pathogens can no longer by itself be taken as an evidence for the presence of autophagosomes and that some other unconventional compartments involving autophagic markers exist. In this regard, it is interesting to note that the PV membrane is derived from the host cell’s plasma membrane, although it is distinct from a phagosomal membrane, as it does not contain host proteins allowing transformation into a mature phagolysosome [17,86]. It is intriguing to note that LC3 (or related proteins) can be recruited to different membrane-bound compartments such as autophagosomes, phagosomes, or directly to parasite-containing vacuoles, yet the factors driving the specificity of this recruitment are unknown.

Regarding the innate immune system it should also be noted that interferon-inducible GBPs are important for the activation of the inflammasome[87], a multiprotein oligomer is responsible for the activation of inflammatory responses that potentially play a role in host resistance to toxoplasmosis [88,89]. As canonical autophagy is involved in the regulation of inflammasome activation[90], it would be interesting to investigate further the interaction between these pathways in the context of *T. gondii* infection.

Moreover, autophagy also has implications for the adaptive immune response, as antigens originating from parasite degradation by this process may be presented by class II molecules of the major histocompatibility complex[91]. This might be important for the cellular and humoral responses to *T. gondii*. However, this needs to be explored further as previous work suggested only ATG5, but not ATG7-dependent canonical autophagy, might promote antigen presentation in the context of *T. gondii* infection[57].

Host autophagy may thus facilitate the optimal regulation of innate immune signaling, the elimination of the parasites, but also the enhancement of antigen presentation in the context of adaptive immunity. Therefore, stimulating this pathway by the use of pharmacological agents is potentially of therapeutic interest for combating infectious diseases[92]. However, although there are extensive preclinical animal model data supporting a potential therapeutic use of autophagy upregulation in the context of several diseases, there are no molecule currently available for use in humans[93]. One of the reasons is that autophagy is a complex process, involved in multiple cellular pathways and maintaining a delicate balance between degradation and biosynthesis; thus stimulating autophagy can have a positive or a negative impact, depending on the context of a given disease or the stage of disease progression.

The case of *T. gondii* infection also illustrates this. For instance, like other intracellular pathogens[94], it seems this parasite has evolved strategies to evade host autophagy machinery-mediated degradation [68,71], or even exploit it as a source of nutrients [80,81]. To divide intracellularly, the parasite has to scavenge some essential nutrients of which the host cell is a valuable source. Interactions between host organelles and the complex interface constituted by the PV limiting membrane[86], facilitate molecular exchanges between host and parasites. By nature, autophagosomes are potential nutrient sources. Interestingly, when not under pressure by the immune system, *T. gondii* can recruit host autophagosomes and even stimulate lipid droplets degradation by lipophagy to gain access to nutrients. However, the parasitic factors that contribute to the recruitment and exploitation of host autophagosomes remain to be identified.

In conclusion, depending on the host organism, the cell type and the type of immune stimulation, the autophagy machinery will have a different impact on the intracellular fate of *T. gondii*. A clearance function of autophagy may enhance pathogen killing in host cells that have been activated for anti-parasitic function, while in permissive host cells *T. gondii* may co-opt the autophagy machinery for its own benefit. This illustrates the remarkable role played by this machinery in the intricate sets of interactions between *T. gondii* and its host cells.
